# Design and Fabrication of an Additively Manufactured Aluminum Mirror with Compound Surfaces

**DOI:** 10.3390/ma15207050

**Published:** 2022-10-11

**Authors:** Jizhen Zhang, Chao Wang, Hemeng Qu, Haijun Guan, Ha Wang, Xin Zhang, Xiaolin Xie, He Wang, Kai Zhang, Lijun Li

**Affiliations:** 1 Changchun Institute of Optics, Fine Mechanics and Physics, Chinese Academy of Sciences, Changchun 130033, China; 2 University of Chinese Academy of Sciences, Beijing 100049, China; 3 Smart Optics Co., Ltd., Changchun 130102, China

**Keywords:** additive manufacturing (AM), 3D printing, microsatellites, lightweight mirror, metal mirror

## Abstract

Microsatellites have a great attraction to researchers due to their high reliability, resource utilization, low cost, and compact size. As the core component of the optical payload, the mirror directly affects the system package size. Therefore, the structural design of mirrors is critical in the compact internal space of microsatellites. This study proposes a closed-back mirror with composite surfaces based on additive manufacturing (AM). Compared with the open-back mirror, it provides excellent optomechanical performance. In addition, AM significantly reduces the intricate mechanical parts’ manufacturing difficulty. Finally, the roughness was better than 2 nm. The surface shape of the AM aluminum mirror reached RMS 1/10λ (λ = 632.8 nm) with the aid of ultra-precision machining technologies such as single-point diamond turning (SPDT), surface modification, and polishing, and the maximum deviation of the surface shape was about RMS 1/42λ (λ = 632.8 nm) after the thermal cycle test, which verified the optical grade application of AM.

## 1. Introduction

The design concept for integrated and lightweight microsatellites was proposed to improve traditional satellites’ reliability and resource utilization. According to the international general classification principle, microsatellites refer to satellites with a weight in the range of 10–100 kg [1]. Turan et al. reviewed the numerous advantages of microsatellites, such as short production cycles, low development, launch costs, high design function density, and mobility [2]., Moreover, optical microsatellites have been widely used in military and space science. Compared with the large aperture space optics, these integrated space-borne payloads have a greater application prospect in comprehensive low-cost earth observation and commercial development [3,4]. However, the internal space of the microsatellites is limited. It is necessary to carry out effective configuration and layout for utilizing the space fully, resulting in high integration for each piece of equipment. At the same time, the package size of the whole configuration is as tiny as possible to reduce the transportation cost, which generally requires the optical system to be compact.

Mirrors are the most critical elements in the optical system. Their aperture sizes and mounting methods directly affect the package size of the optical payload, and their optical and structural characteristics play a critical role in the performance of the entire system [5]. The excellent processing characteristics of metal materials make it easy to integrate optical and mechanical elements. Therefore, the advantages of metal mirrors can meet the requirements of microsatellites perfectly, and relying on modern ultra-precision machining technology, metal mirrors can be processed to ideal surface quality [6,7]. In the previous work by the authors, an off-axis aluminum (Al) mirror for visible-light imaging was designed and manufactured [8]. Actual optomechanical integrated manufacturing can be realized by optimizing the whole process chain of design, manufacturing, testing, and assembly. Xie et al. fabricated an all-Al imaging telescope, verifying that the free-form Al mirror benefited from a compact package size and a wide field of view for optical systems [9]. Additionally, the optical system could achieve the optimal athermal effect when the mirror and the mounting structures were made of the same materials. Liu et al. optimized and manufactured an Al mirror with an aperture of 160 mm [10]. According to the test results, the error budget showed that the mirror kept a fine surface shape at low temperatures. Therefore, Al mirrors have good prospects for low-temperature applications. According to Vukobratovich’s calculation, the manufacturing cost of metal materials for the mirror is only about 50%, compared with ceramics, glass, and other materials [11]. Thus, the all-Al configuration is also a cost-effective product, with the advantages of athermalization and short lead time.

However, more and more deficiencies of the conventional lightweight mirrors have been highlighted in many cases to dates, such as the limitation of flexible structures, quilting deflection, and even the stress-induced surface deformation of the coating [12,13]. Especially in the compact optical systems of microsatellites, it is challenging for conventional mirrors to achieve the trade-off between lightweight and mechanical properties in such a limited space. The closed-back mirror can improve mechanical performance while maintaining the lightweight rate, but it is challenging to fabricate through conventional processing methods [14]. Fortunately, with the continuous advancement of metal additive manufacturing (AM) technology, the form of layer-by-layer printing dramatically reduces the difficulty of manufacturing intricate mechanical parts. Furthermore, AM metal parts have good fatigue properties [15]. Zhou et al. explored the fatigue properties of as-built AlSi_10_Mg parts with curved surfaces, which provides a good reference for the optical spheric and aspheric mirrors [16]. AM is a promising solution to optimize mass and dimensional stability to a higher level than conventional machining.

Nevertheless, it is also precisely because of the layer-by-layer printing that the as-built AM parts are arduous for obtaining optical surfaces. Extensive research has been carried out to develop the new-generation metal mirror based on AM. Sweeney et al. provided case studies utilizing multiple metal AM technologies for mirror designs and discussed the findings, such as stress relieving and isotropy of mechanical properties [17]. Hartung et al. designed and manufactured the Voronoi AM mirror for visible-light level application [18]. Robert et al. presented and incorporated the circular lattice into the mirror for a CubeSat telescope [19]. Yan et al. designed an assembly-level topology optimization of Al mirrors based on AM, which improved the efficiency of assembly and integration [20]. Moreover, the modeling rules are also essential for the manufacturing accuracy of precision optical elements. Sara et al. proposed design rules for improving the accuracy of selective laser melting (SLM) manufacturing using benchmark parts [21]. Table 1 gives a comparison study with the mentioned literature. While other work has shown successes in AM mirror preforms that were post-polished, discussions on the design and manufacturing methodology leveraging AM have not been discussed in detail.

Different from other research, this paper aims to balance the lightweight, optical, and structural performances of the large aperture mirror in the limited space for microsatellites. An Al mirror with composite surfaces based on AM is proposed for low material cost and compact design. Compared with the open-back scheme manufactured by conventional machining, the closed-back mirror provides better optomechanical properties through finite element analysis (FEA). Then, a complete manufacturing process of AM metal mirror, such as AM for mirror blank, single point diamond turning (SPDT), surface modification, and polishing, is described in detail. Then, the paper examines the surface-shape accuracy and the infrared imaging requirements. Finally, a discussion of achievements and future work closes this paper. 

## 2. Optical System and Structural Design Input

**Figure 1:**
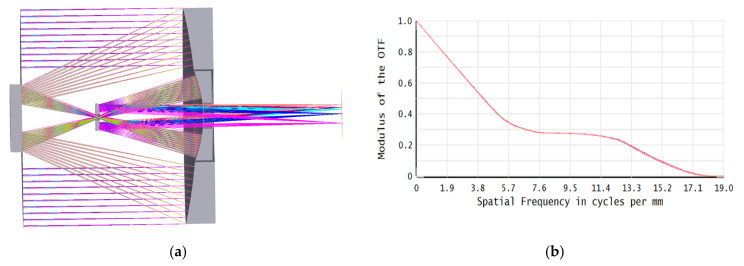
(**a**) Light traces of the optical system; (**b**) MTF image.

Due to the constraints of microsatellite platform resources, reducing the weight and volume has become the focus of optical payload design and optimization. A compact coaxial reflective optical system with four mirrors is presented in this paper, as shown in Figure 1a. It is an F/10 optical system with a focal length of 1750 mm working in the infrared band. All four mirrors are aspheric surfaces. After reflection by the primary mirror (PM) and the secondary mirror (SM), the light passes through the central hole of the quaternary mirror (QM) to arrive at the tertiary mirror (TM). The central hole is the field stop of this optical system. Finally, the light forms a perfect image on the detector plane after four times of reflection, and the modulation transfer function (MTF) image is given in Figure 1b.

**Figure 2:**
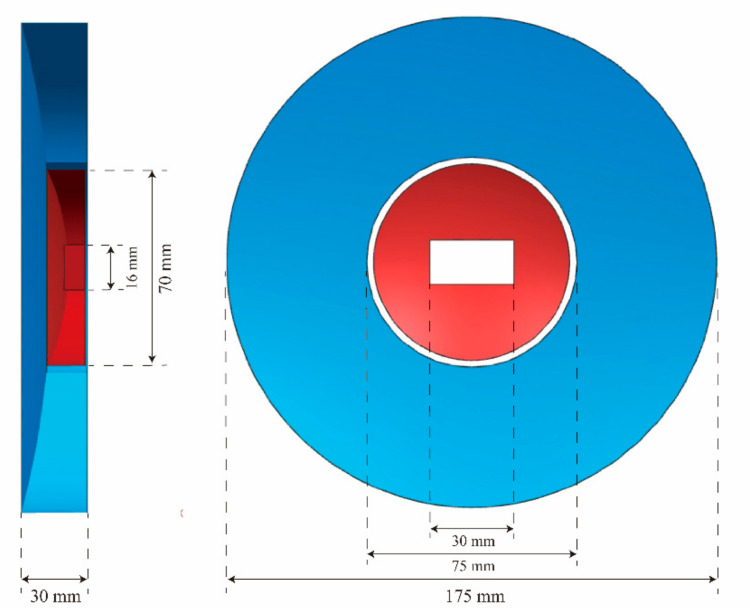
Schematic diagram of the relative position for the PM and the TM.

Figure 2 shows the relative position of the PM and the TM. The effective aperture of the PM and the TM is 175 mm and 70 mm, respectively. The size of the PM center hole is 75 mm. There is a 30 mm × 16 mm rectangular hole in the center of the TM resulting from the field stop. In the initial design, the distance difference between the PM’s center hole and the TM’s outer circle was 0.2 mm in the optical axis direction. Considering the environmental conditions in which the optical system is located, the structural elements should be as light as possible to ensure appropriate structural performance. Therefore, the integrated scheme of the PM and TM was adopted with a lightweight rate of 70%. Based on providing an effective aperture, the clear aperture of the TM was increased appropriately, and the center hole of the PM was reduced. Optical design optimization allows the PM and TM edges to be fused into a continuous sectional curve. This integrated scheme minimizes the difficulty of the system assembly and wavefront aberration correction. The parameters and specifications for the mirror with compound surfaces are given in Table 2.

## 3. Structural Design and Finite Element Analysis

### 3.1. Structural Design for Mirrors with Compound Surfaces

**Figure 3:**
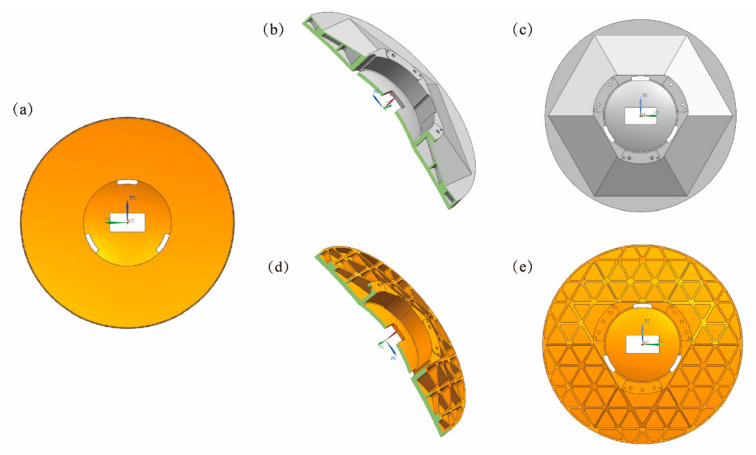
Schematic diagram of mirror models: (**a**) front view of the mirror; (**b**) half-section of the closed-back mirror; (**c**) back view of the closed-back mirror; (**d**) half-section of the open-back mirror; and (**e**) back view of the open-back mirror.

The PM and the TM were integrated into a whole during structural design based on their position relationship. PM is the component with the largest package size in the radial direction to reduce the system volume. In addition, three through-holes are evenly distributed on the mirror to pass through the support structures (Figure 3a). As mentioned, this structure is more compact, which is conducive to reducing the weight and volume of the system. The manufacturing process guarantees the relative position of the PM, the TM, and the rectangular hole, which simplifies of the optical system assembly. It can also reduce manufacturing time and cost. Conventional glass and SiC mirrors are made of hard and brittle materials, so it is difficult to realize the processing of intricate mechanical parts. Furthermore, because of the optical processing technology of grinding and polishing, the relative position relationship between the two mirrors cannot be accurately guaranteed [22]. Therefore, an all-Al alloy design was adopted, and the PM and the TM share one mirror substrate.

The open-back mirror and the closed-back mirror were designed in this paper. The closed-back structure has one more back plate of 2 mm than the open-back structure. Therefore, it is also referred to as the sandwich mirror. Because of the AM, the manufacturing difficulty of the complex sandwich lightweight structure is significantly reduced. Daniel Vukobratovich found that the sandwich mirror has the best mirror efficiency, defined as the total mirror height divided by the mechanical deflection [23]. The sandwich mirror places most mass in the face and back plates, with as little mass as possible in the shear core. It provides high stiffness and efficient bending when the mass of a structure is distributed as far as possible from the neutral or bending axis. The mirrors inside are supported by triangular geometry, which can trade off overall stiffness and lightweight rate. The average thickness of the ribs is about 1.5 mm, and the mirror surface thickness is 4 mm. The half-sectional and rear views of the two mirrors are shown in Figure 3. Next, FEA (modal analysis and gravity deformation analysis) is carried out to compare the two structural schemes’ comprehensive optomechanical performance and verify whether they can be applied in engineering.

### 3.2. Finite Element Analysis

#### 3.2.1. Modal Analysis

The modal frequency and mass are negatively correlated as follows:

(1) $$\omega =\sqrt{\frac{k}{m}\;},$$

where ω is the modal angular frequency, k is the rigidity coefficient, and m is the mass. Thus, the masses of the two mirrors were adjusted to be equal to compare the mechanical properties accurately [24]. Since the closed-back scheme has a back plate, the mass is heavier under the same conditions. The surface thickness of the closed-back scheme was reduced (~1 mm) until the equal group. Then, the modal analysis of the mirrors was carried out by fixing the six mounting surfaces.

**Figure 4:**
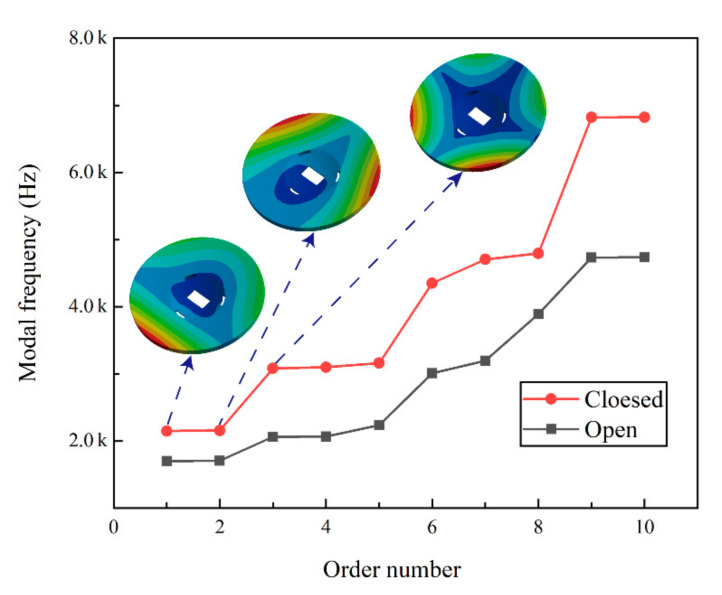
Modal analysis results.

The curves of the first 10-order modal frequencies for the two structural schemes are shown in Figure 4. At the same time, the total deformation of the first three-order for the closed-back scheme is also given. It can be seen intuitively that all modes of the closed-back mirror are higher than those of the open-back mirror. Table 3 shows the specific data of each order mode for the two schemes. The first-order modal frequency of the closed-back scheme is 2149 Hz, and that of the open-back scheme is 1703 Hz; the 10-order modal frequencies of the two schemes are 6823 Hz and 4876 Hz, respectively. Therefore, the overall stiffness of the closed-back scheme is excellent.

#### 3.2.2. Gravity Deformation Analysis

**Figure 5:**
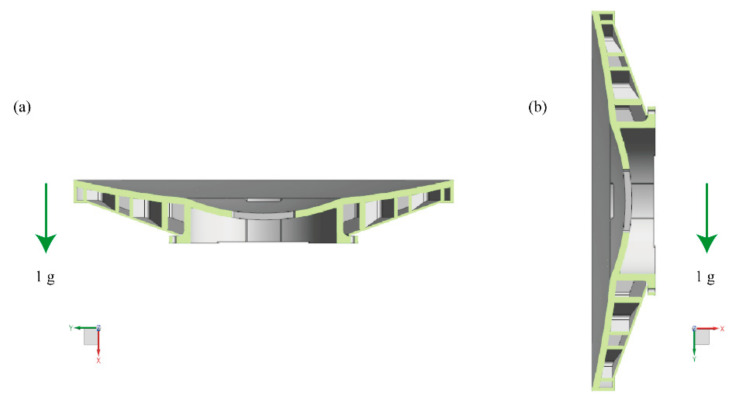
Directions of gravity deformation analysis: (**a**) axial gravity; (**b**) radial gravity.

**Figure 6:**
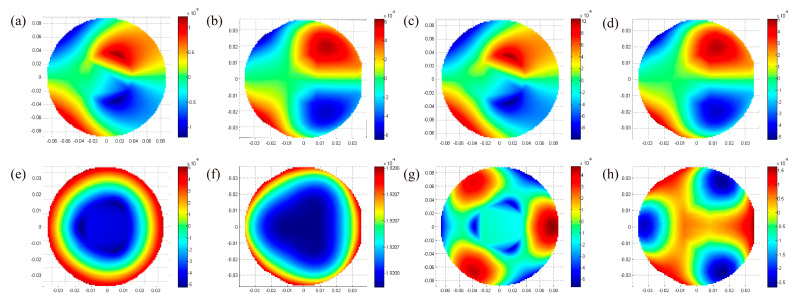
Surface-shape-fitting nephograms after gravity deformation analysis: (**a**) open-PM-radial; (**b**) open-TM-radial; (**c**) closed-PM-radial; (**d**) closed-TM-radial; (**e**) open-PM-axial; (**f**) open-TM-axial; (**g**) closed-PM-axial; (**h**) closed-TM-axial.

Both mirrors are rotationally symmetric, and the gravity deformation analysis was performed under the axial and radial conditions (Figure 5) [25]. Surface-shape-fitting nephograms after gravity deformation analysis are given in Figure 5. It can be concluded from the nephograms that there are no significant differences in the radial deformation of the two schemes. However, the difference in axial deformation is noticeable. As mentioned, for beams distributed in the sandwich mirror, most of their mass is far from the bending axis. It is an efficient structure in bending in which relatively little mass is placed in the web of the beams. Thus, the surface deformation caused by the axial shear force can be reduced. The fitting data corresponding to Figure 6 is shown in Table 4. The radial deformation value of the closed-back scheme is about 20% smaller than that of the open-back scheme. The axial deformation value of the closed-back scheme is about 15–25%, significantly less than that of the open-back scheme. Consequently, the closed-back mirror has a pronounced effect on improving the mirror’s gravitational deformation.

## 4. Mirror Fabrication

**Figure 7:**
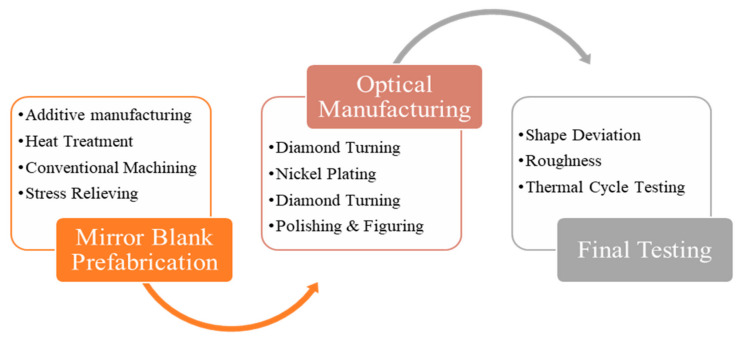
Processing chain for an AM metal mirror.

As shown in Figure 7, the processing chain for AM metal mirrors was divided into three stages. The first stage is the mirror blank prefabrication stage. After the structural design, the mirror blank is printed via metal AM technology. Stress relieving through densification and heat treatment prepares for the next step. Optical manufacturing is to obtain a high-quality optical surface through ultra-precision processing technologies. Finally, the surface quality and thermal performance of mirrors were tested.

### 4.1. Mirror Blank Prefabrication

#### 4.1.1. AM for the Mirror Blank

Laser powder bed fused (LPBF) is a specific kind of metal AM method that generates parts out of powder on a layer-by-layer principle. It converts the laser energy into heat energy to make parts form [26]. The laser focuses the spot to tens of micrometers, and the power density is more than 5.0 × 10^6^ W/cm^2^. A high-energy laser beam completely melts powder under a protective atmosphere, it can avoid shrinkage cavity, porosity, and inclusion to improve the density. Laser absorptivity in AM is essential, showing the parts’ quality and transition from keyhole to conduction mode [27]. Rapid cooling inhibits grain growth and segregation of alloy elements, resulting in refined grains and uniform element distribution [28]. At the same time, LPBF also inhibits grain growth to form fine grain strengthening. Its mechanical properties, such as tensile strength, are better than castings and even reach the level of forgings. The Al alloy powder was selected as AlSi_10_Mg, where the density was 2.67 g/cm^3^, the modulus of elasticity was 69 GPa, and the Poisson’s ratio was 0.33. AlSi_10_Mg is a hardenable Al-based alloy. Cooperating with LPBF, it is applicable for thin-walled components with complex geometries. LPBFed AlSi_10_Mg is highly suitable for processing and characterized by good resistance in corrosive atmospheres and high electrical conductivity [29]. The combination of high achievable strengths while maintaining dynamic load capacity enables it to be used for highly stressed parts. Therefore, the high density, high printing precision, and excellent mechanical properties make LPBF suitable for AM metal mirror manufacturing [30].

**Figure 8:**
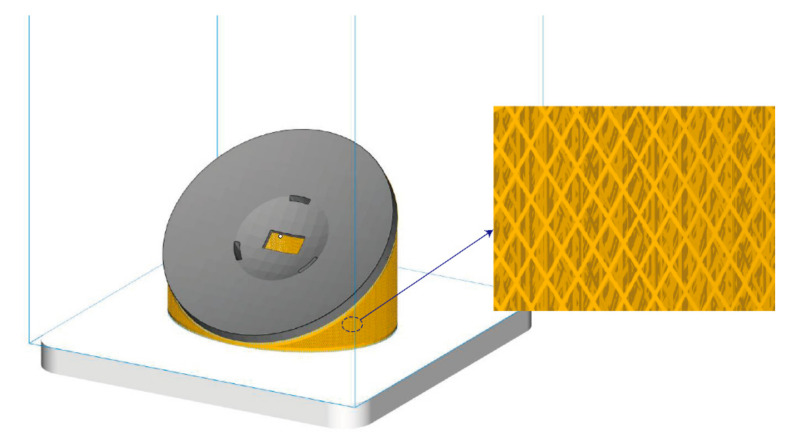
Visualization of support structures.

The spherical powder with the size of 20–30 μm was selected, and the compactness of the printed parts is better than 99.5% to ensure that its fluidity meets the requirements. SLM Solutions 280 is chosen for the mirror blanks forming. It provides a 280 × 280 × 365 mm³ build room and patented multi-beam technology. In addition, the patented bidirectional powder coating helps reduce the manufacturing time of individually manufactured metal parts. The thickness of the powder layer is 25 μm, and the scanning speed is 980 mm/s. Argon gas with an oxygen mass fraction of less than 0.1% was used to protect the whole printing process and prevent metal oxide formation. For ensuring good fixation of the mirror to the building platform and preventing collapse and deformation during printing, the complex support structures were designed [31]. The mirror blank is placed at an angle of about 40°, as shown in Figure 8. The self-supporting diamond structures were selected as the support structures with a thickness of 0.2 mm and an angle of 120°, which can save powders and cost. In addition, the removal difficulty of the support structures is reduced via computer numerical control machining (CNC) and wire cutting.

**Figure 9:**
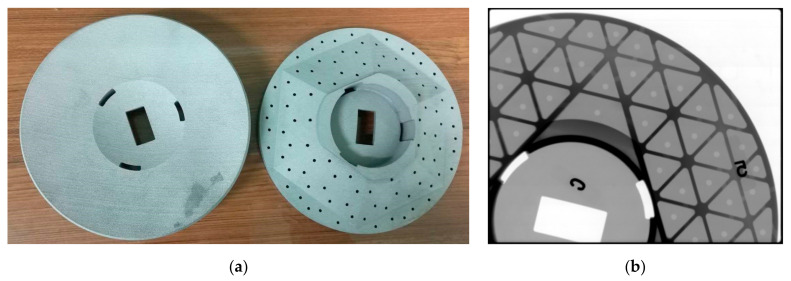
(**a**) AM mirror blanks after cleaning; (**b**) X-ray inspection image for the AM mirror blank (the letter and number are locating datum).

The final LPBFed mirror blanks are shown in Figure 9a. Technological holes were added to the design model to facilitate the cleaning of residual powders during the AM process. These technological holes are mainly located on the side wall of the stiffeners between the mirror back and the lightweight cells. All the enclosed areas inside the mirror body through these holes relate to the outside. These holes have little impact on the mirror’s overall stiffness and mechanical characteristics due to the small sizes and discrete distribution. Figure 9b is an X-ray test image for a 3D-printed mirror blank. The positions of technological holes and lightweight ribs can be seen clearly.

#### 4.1.2. Heat Treatment and Densification

As a result of the unique process of metal AM, the residual stress in the formed parts is enormous. Therefore, heat treatment for stress relieving is the first post-treatment step after forming. In the LPBF process, the powders are not completely melted, especially the Si particles, leading to micropores in the formed mirror. Therefore, densification treatment is also required to eliminate pore defects. Hot isostatic pressing (HIP) is a special heat treatment for reducing the porosity of AM mirrors. In the HIP, the mirror blank was placed in a furnace with a temperature of ~550 °C for 2 h. At the same time, the furnace was filled with argon and pressurized at ~110 MPa [32,33]. Then, the isostatic pressing was applied to the material in all directions until the mirror blank cooled.

#### 4.1.3. Conventional Machining and Thermal Cycling

**Figure 10:**
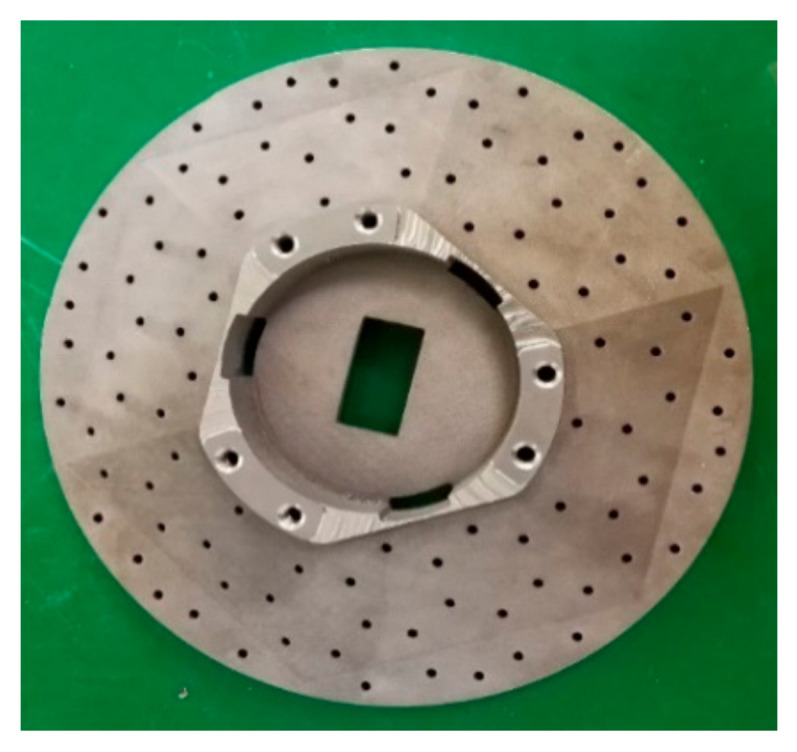
The mounting benchmark after CNC.

After densification, the structural elements with specific accuracy requirements need to be finished by traditional processing methods, including the mirror surface, the structural reference plane, the screw hole, and the pinhole. The processing number in this process was controlled by 1 mm. The mounting benchmark after CNC is shown in Figure 10, and the rough (1 mm machining allowance), semi-finishing (0.5 mm machining allowance), and finishing machining (0.1 mm machining allowance) were carried out by CNC until the surface roughness was about 1.6 μm.

Thermal cycling and the aging treatment are the processes to improve mechanical properties and reduce the residual stress of mirror blank. Thermal cycling stabilizes components by repeatedly alternating the part temperature between approximately low-temperature and high-temperature conditions at a controlled rate of a few degrees per minute. This obtains a measure of stress relaxation for those small stresses introduced in the rough machining and finishing processes. Heat treatment was carried out once for each machining. The thermal cycling process first placed the AM mirror in liquid N_2_ and held it for 30 min. It was then heated to 23 °C for 30 min and heated to 160 °C for 30 min. Finally, the mirror was cooled to 23 °C. The thermal cycling temperature range was −180 °C to +160 °C, with the temperature changing rates less than 2.0 °C/min.

### 4.2. Optical Manufacturing

#### 4.2.1. SPDT before Plating

**Figure 11:**
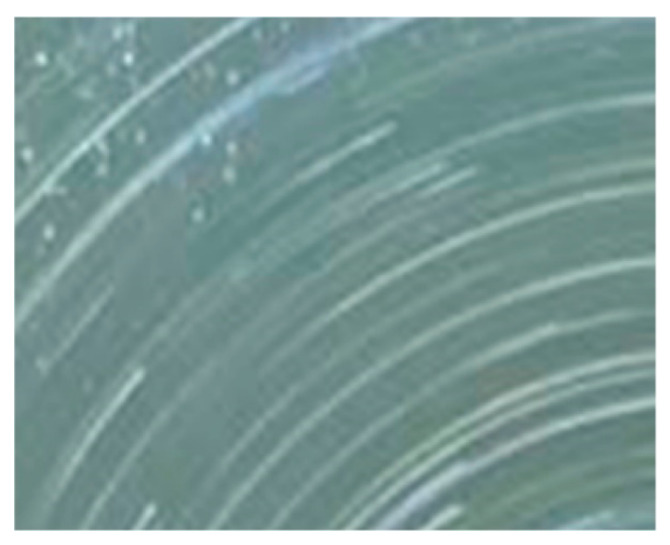
Mirror blank surface under white light.

The mirror blank was fixed on the main shaft through a particular tooling disk. After the first round of SPDT, the flatness of all mounting surfaces was better than 1.0 μm. Moreover, the mirror surface was poor. These pits and turning scratches can be seen more clearly under white light (Figure 11). The insufficient density of parts causes dimples during AM. When the diamond tip passes through the pit in the high-speed turning process, it produces vibration and leaves fine scratches. These macroscopic surface defects will cause strong scattering of the imaging light and seriously affect the system’s signal-to-noise ratio. Thus, surface modification is necessary for optical applications.

#### 4.2.2. Surface Modification and Second Round SPDT

**Figure 12:**
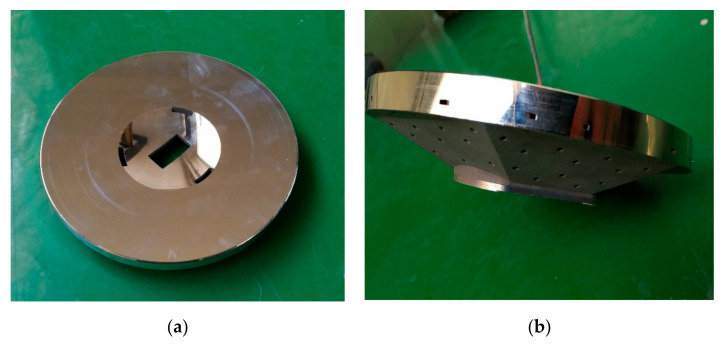
The mirror after surface modification: (**a**) front view; (**b**) side view.

Electroless nickel (Ni), a coating NiP alloy on the Al-Si substrate by electrochemical deposition, is the most common way for surface modification of Al mirrors. For the homogenous layer composition, the Ni content, pH value, and bath temperature of the electrolyte must be constant during the deposition process. It is necessary to monitor and control them in situ. At constant bath temperature and Ni content, the alloy’s final phosphorus (P) contents are different, which is affected by pH value [34]. The P content is the most significant parameter for tailoring the mechanical and thermal properties, such as machining characteristics and coefficient of thermal expansion (CTE). The NiP layer becomes amorphous and isotropic at over 10.5 wt %. Moreover, the tool wear is serious during SPDT for the NiP layer when P content is less than 10.5 wt % [35]. The final range of P content is limited to 10.5 wt % to 14 wt %. The mirror, after surface modification, is shown in Figure 12. Then, the second round of SPDT was carried out on the NiP surface with the machining allowance of 1.0 μm.

#### 4.2.3. Polishing and Figuring

**Figure 13:**
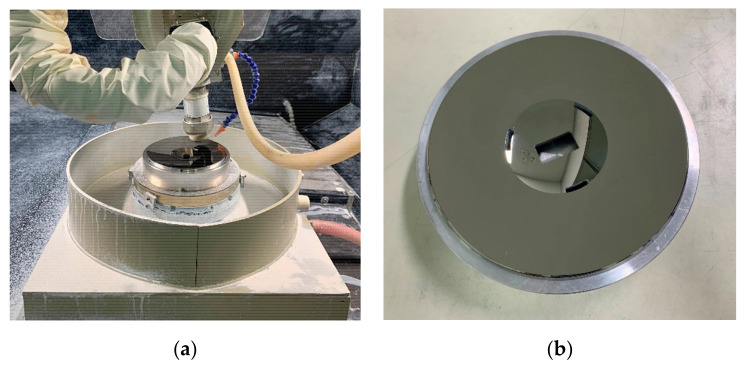
(**a**) The mirror on the OptoTech polishing machine; (**b**) the final mirror in the tool disk.

Polishing and figuring were adopted to correct mid- and high-frequency and low-frequency errors of the mirror. The mid- and high-frequency errors are mainly the periodic tool marks during SPDT. Above all, these tool marks have a strong scattering effect on the imaging light and affect imaging quality. Additionally, the low-frequency errors are mainly the surface-shape deviation of the mirror surface. An OptoTech machine was utilized for polishing with a CNC asphalt head (Figure 13a) [36]. After polishing, the turning tool marks of the mirror surface were suppressed dramatically. The final mirror after polishing is shown in Figure 13b. Although ion beam figuring (IBF) positively affects the surface processing of metal mirrors, its time and processing cost are high [37]. Therefore, IBF will be used to verify the influence on the mirror surface shape in the next iteration.

### 4.3. Final Testing

#### 4.3.1. Surface-Shape Deviation and Roughness Testing

**Figure 14:**
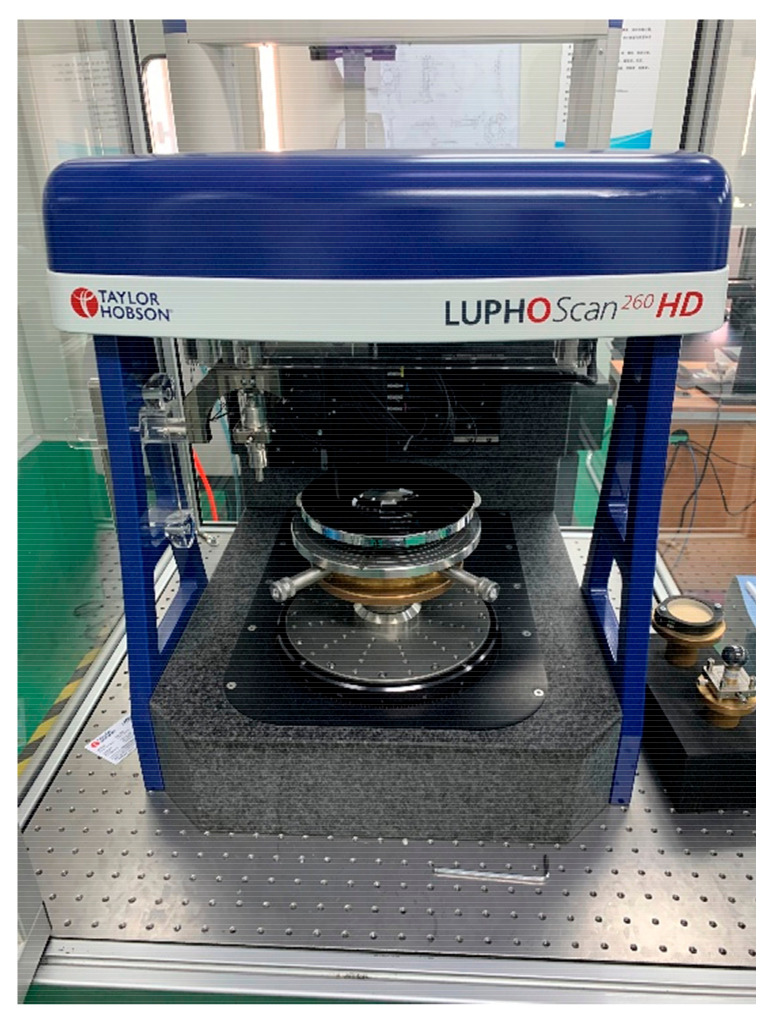
The mirror is testing on LUPHOScan.

Because of the aspherical optical prescription, a 3D profilometer-Taylorhobson-LUPHOScan (Taylor Hobson Ltd., Leicester, UK) was adopted for the surface-shape testing of the whole process (Figure 14). It is designed to perform ultra-precision 3D-form measurements for rotationally symmetric spherical and aspheric surfaces. LUPHOScan is even capable of free-form surface measurements easily, which dramatically reduces the surface deviation testing difficulty. The Results from non-contact measurements show that it is ideal for applications of high-precision requirements.

**Figure 15:**
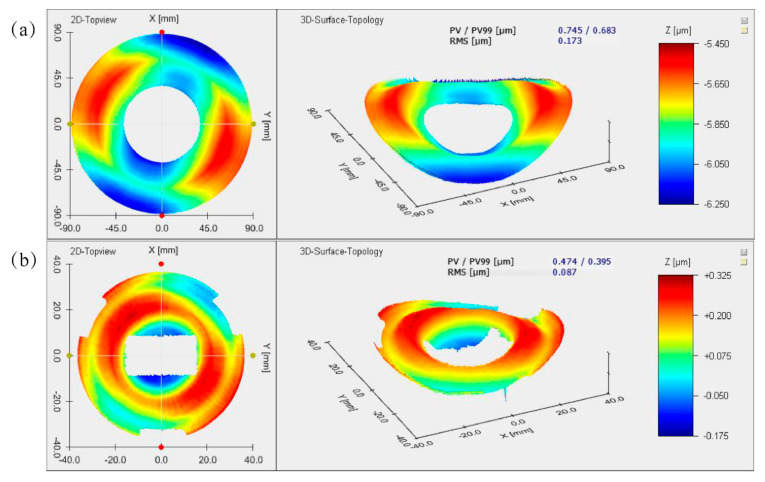
Surface shape deviation after the second Round SPDT: (**a**) PM; (**b**) TM.

**Figure 16:**
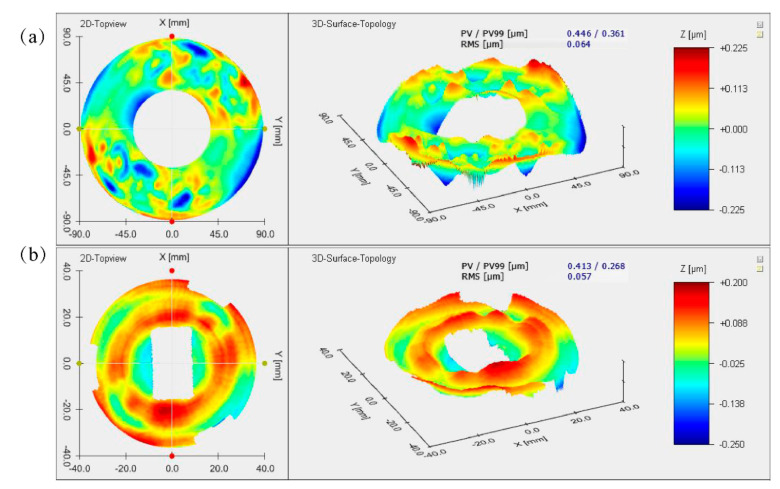
Surface shape deviation after polishing: (**a**) PM; (**b**) TM.

The form error testing was not carried out after the first round of SPDT due to the poor surface quality. Figure 15 and Figure 16 show the surface-shape testing results after the second round of SPDT and polishing. After surface modification and the second SPDT, the surface shape of the PM and the TM is RMS 173 nm and RMS 87 nm, respectively; after polishing, the surface shape of the PM and the TM is RMS 64 nm and RMS 57 nm, respectively.

**Figure 17:**
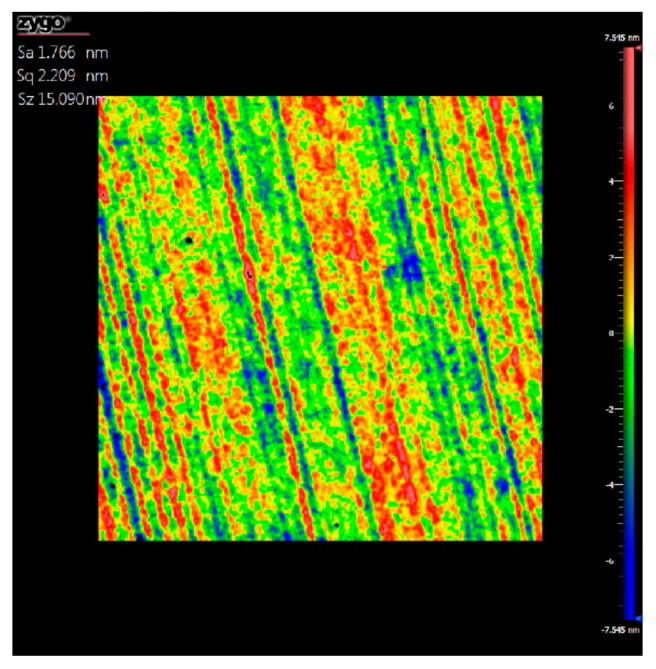
Roughness testing results using white light interferometry.

Subsequently, local surface roughness was measured using white light interferometry to assess the homogeneity and distribution of contamination and defects. The result shows that the final roughness was about 1.76 nm, less than 2 nm (Figure 17). Summarily, the surface shape and roughness both meet the parameters and infrared imaging requirements given in Table 2. 

#### 4.3.2. Thermal Cycle Testing

**Figure 18:**
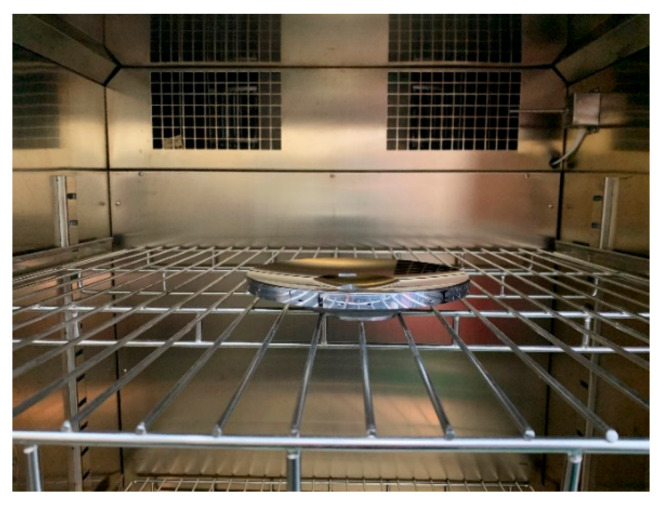
The AM mirror in the test furnace.

Generally, the dimensional stability of metal mirrors must be kept within the range of 10^−6^, amounting to the dimension variation at the meter scale being controlled at the micrometer scale. For AM mirrors, dimensional stability is more critical due to the residual stress caused by the unique forming process. Therefore, it is necessary for the thermal cycle test of AM metal mirrors. Figure 18 shows the AM mirror in the testing furnace. Then, the stability in the extreme environment and whether it meets the practical engineering requirements is verified. The maximum temperature range of the system during operation and storage is −40–60 °C. Thus, this thermal cycle testing set the high-temperature and low-temperature conditions at −50 °C and +70 °C, respectively. The cycle was carried out three times with a temperature change rate of 1.0 °C/min, and the holding time was an hour. 

**Figure 19:**
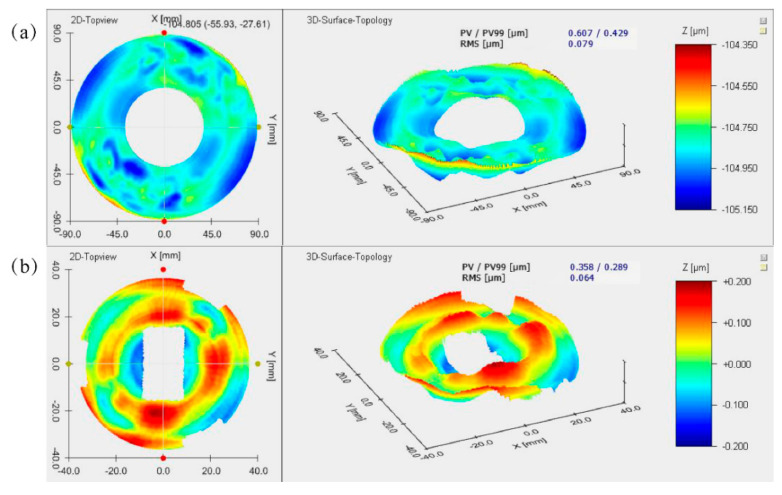
Surface-shape deviation results after the thermal cycle test: (**a**) PM; (**b**) TM.

After the thermal cycle testing, the mirror surface-shape deviations are shown in Figure 18. The surface shape of the PM (Figure 19a) and the TM (Figure 19b) is RMS 79 nm and RMS 64 nm, respectively. Table 5 summarizes the mirror surface shape under different conditions. Compared with the polished surface, the change of the PM surface shape is RMS 15 nm; the change of the TM surface shape is only RMS 7 nm, which conforms to the basic requirements for optical mirror surface quality, and the slight change in the surface quality could return to its original state. After the thermal cycle testing, the maximum deviation of the surface shape is 15 nm, about RMS 1/42λ (λ = 632.8 nm). It proved that aging and heat treatment is effective and verified AM’s optical grade application.

## 5. Conclusions and Future Work

This study looked to develop an AM mirror with compound surfaces. The integrated mirror is designed for use in microsatellites, greatly reducing space occupancy, cycle time, and launch cost. Through FEA, it was found that the performance of the closed-back scheme is better than that of the open-back scheme. The closed-back mirror is printed via LPBF technology. After SPDT, surface modification, and polishing, the surface-shape accuracy of the TM and the PM reached RMS 57 nm and RMS 64 nm, respectively, and the roughness was better than 2 nm. Finally, thermal testing was carried out to further verify the mirror’s dimensional stability. Results showed that the surface shape changed within RMS 15 nm, which reached the optical stability requirements.

This work has been successful so far, although the surface shape of the mirror is only about 1/10λ (λ = 632.8 nm). It can be seen from the thermal cycle testing results that the maximum surface-shape change is about 1/40λ (λ = 632.8 nm) which explains that the aging treatment is effective. Due to the time limit, IBF was not performed. Subsequently, the effect of IBF on the AM mirror surface will be tested to achieve a high-precision surface shape that meets the visible-light-level application. In addition, the traditional isogrid design was adopted for the mirror design. More advanced structures will be developed to take advantage of the free-form design of AM and trade off the performances well in future.

## Figures and Tables

**Table 1:** Comparison with the other literature.

Authors	Aperture (mm)	Material	Highlight
Sweeney et al. [17]	75–150	AlSi_10_Mg etc.	Process exploration and validation
Hartung et al. [18]	76	AlSi_40_	Novel lightweight structure design
Robert et al. [4]	72	AlSi_10_Mg	Lattice mirror design for CubeSat
Yan et al. [21]	58	AlSi_10_Mg	Assembly-level topology optimization and integration design
This study	175	AlSi_10_Mg	Large aperture and integration design with compound surfaces

**Table 2:** Parameters and specifications for the mirror with compound surfaces.

Parameter	Specification (PM)	Specification (TM)
Optical prescription	Aspheric surface	Aspheric surface
Radius of curvature	396 mm	98 mm
Effective Aperture	175 mm	70 mm
Form error	Better than RMS 1/10λ (λ = 632.8 nm)	
Roughness	Better than 2 nm	
First-order modal frequency	2000 Hz	
Lightweight rate	70%	

**Table 3:** Modal analysis data.

Order Number	Open-Frequency (Hz)	Closed-Frequency (Hz)
1	1703.4	2149.6
2	1705.3	2157.2
3	2064.1	3082.5
4	2068.1	3099.7
5	2236.1	3160.1
6	3011.3	4351.0
7	3195.2	4705.6
8	3891.6	4795.5
9	4733.5	6821.2
10	4736.1	6823.9

**Table 4:** Surface-shape-fitting results after gravity deformation analysis.

	Open-PM	Open-TM	Closed-PM	Closed-TM
Radial (P.V. nm)	22.8	12.9	20.6	10.5
Radial (RMS nm)	5.4	3.3	4.6	2.6
Axial (P.V. nm)	58.3	18.6	10.5	4.4
Axial (RMS nm)	16.7	4.0	2.5	1.0

**Table 5:** Summary of mirror surface shape under different conditions.

Process Steps	PM-RMS (nm)	TM-RMS (nm)
First Round SPDT	/	/
NiP + Second Round SPDT	173	93
Polishing	64	57
After the thermal cycle test	79	64

## Data Availability

Not applicable.
